# Association of tyrosine hydroxylase expression in brain and tumor with increased tumor growth in sympathectomized mice

**DOI:** 10.1186/s13104-021-05507-w

**Published:** 2021-03-10

**Authors:** R. Gomez-Flores, I. Gutierrez-Leal, D. Caballero-Hernández, A. Orozco-Flores, P. Tamez-Guerra, R. Tamez-Guerra, C. Rodríguez-Padilla

**Affiliations:** Laboratorio de Inmunología y Virología, Departamento de Microbiología e Inmunología, Facultad de Ciencias Biológicas, Universidad Autónoma de Nuevo León, Apartado postal 46 F, San Nicolás de los Garza, 66451 NL México

**Keywords:** Sympathectomy, Denervation, 6-OHDA, Lymphoma, Tumor burden, Body weight, Adiposity, Cytokines

## Abstract

**Objective:**

Lymphocytes express tyrosine hydroxylase (TH), the rate-limiting enzyme for the synthesis of dopamine, norepinephrine and epinephrine. This suggests a broader role for cathecholamines in lymphocyte function, as well as the potential secretion of catecholamines by tumors of lymphoid origin. Our aim was to evaluate the expression of *Th* by murine lymphoma cells in an in vivo mouse model. For this, L5178Y-R lymphoma cells were implanted in nerve-intact and sympathectomized male BALB/c mice. Relative *Th* gene expression in tumor and brain was determined by quantitative PCR. Body composition, tumor volume, and plasma TH1/TH2/TH17 cytokines were also evaluated as markers of tumor-host condition and anti-tumor immune response in absence of adrenergic innervation.

**Results:**

We found a significant (*p* = 0.045) 3.3-fold decrease of *Th* gene expression in tumor and a non-significant (*p* = 0.60) 6.9-fold increase in brain after sympathectomy. Sympathectomized mice also showed a significant increase in tumor mass at days 18 (*p* = 0.032) and 28 (*p* = 0.022) and increased interscapular fat (*p* = 0.04). TH1/TH2 and TH17 cytokines levels in plasma from sympathectomized tumor-bearing mice were not different from control mice.

**Conclusion:**

The L5178Y-R lymphoma does not express *Th* during in vivo progression.

## Introduction

Tyrosine hydroxylase (TH) is an enzyme involved in the synthesis of the catecholamines dopamine, norepinephrine (NE), and epinephrine. It is mainly expressed by central and peripheral nervous tissues and adrenal glands [1]. TH has several important physiological roles, including autonomic reflexes, behavior, cognition, and endocrine functions; its expression is regulated at transcriptional, translational, and post-translational levels in response to internal and external cues [2]. Evidence of its expression in lymphocytes [3] may indicate a broader role for catecholamines in immunomodulation. Furthermore, *Th* expression and catecholamine biosynthesis are modulated in splenic T- and B- cells in Sprague Dawley rats under immobilization stress [4], indicating peripheral modulation by the sympathetic central nervous system.

The potential of lymphocytes to up-regulate the *Th* gene and catecholamine biosynthesis suggests that tumors of lymphoid origin secrete catecholamines.

Tumors are known to release hormones, neurotrophic factors, inflammatory cytokines, and chemokines [5], which act in a paracrine and autocrine fashion. Catecholamines are synthetized and secreted by tumors from nervous tissues, including pheochromocytomas, as well as by paraganglionar and carcinoid tumors. However, there is no evidence of catecholamines synthesis by tumors from non-nervous tissues such as lymphomas. In a previous work, we observed that tumor burden was associated with a significant increase in plasmatic norepinephrine in L5178Y-R lymphoma-bearing mice that was independent of adrenergic activation by stress [6], thus, we hypothesized that L5178Y-R lymphoma cells may be expressing *Th* and secreting NE, contributing to the observed increase in NE circulating levels. The aims of the present study were to assess the expression of the *Th* gene in the L5178Y-R lymphoma model, its association with adrenergic innervation, and changes in body composition, tumor volume, and antitumor cytokines response. This work derives from our research on the role of adrenergic signaling in cancer-associated cachexia.

## Main text

### Methods

#### Animals

Ten BALB/c male mice aged 10 to 12 weeks, with body weight ranging from 26 to 28 g at the beginning of the experiment were provided by the institution's animal facility. This number was reduced to nine when one tumor-bearing mouse died after the administration of 6-OHDA. Animals were kept in pathogen- and stress-reduced micro-ventilated cages, five animals per cage, under a 12-hr light–dark cycle (light phase, 06:00–18:00 h) at 22 °C room temperature and 45% relative humidity. Animals were maintained with water and food ad libitum, woodchip for bedding, plastic pipes, and toilet paper rolls for nesting and environmental enrichment. The experiment complied with Mexican regulation NOM-062-ZOO-1999 and was reviewed and approved by the Internal Committee for the Care and Use of Experimental Animals with number CEIBA-2017–004. The L5178Y-R lymphoma is a transplantation tumor model; standard ethical guidelines on the welfare and use of animals in cancer research [7] were followed. Animal well-being was assessed daily. Tumors that were ulcerated, infected, necrotic or larger than 200 mm^3^, or 15–20% of body weight loss were considered the humane endpoints.

#### Tumor cell line

L5178Y-R tumor cell line (mouse DBA/2 lymphoma) was purchased from The American Type Culture Collection (Rockville, MD) and maintained in culture flasks with RPMI 1640 medium supplemented with 10% FBS, 1% L-glutamine, and 0.5% penicillin–streptomycin solution, at 37 °C, in a humidified atmosphere of 5% CO_2_. The cellular density was kept between 10^5^ and 10^6^ cells/mL.

#### Chemical sympathectomy and tumor implantation

**Fig. 1:**
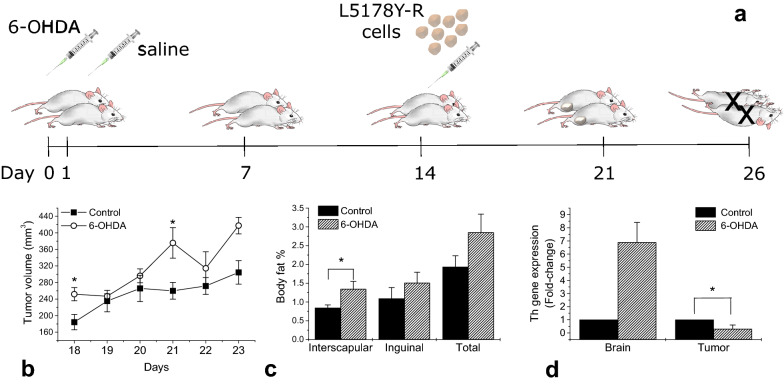
**a** Experimental timeline, a group of mice (n = 4) was chemically sympathectomized with 2 doses of 6-OHDA i.p., the control group (n = 5) received saline i.p., 14 days after sympathectomy, L5178Y-R cells were inoculated in the right flank of control and sympathectomized animals. After mice euthanasia, tissues were surgically excised and stored at − 80 ºC until analysis (**b**) tumor volume progression (**c**) interscapular and inguinal fat wet weight (**d**) Relative gene expression of *Th* was calculated by the 2^−ΔΔCT^ method. Data represent the mean ± SEM of 4–5 animals from a representative experiment. Resulting values were analyzed with the Student *t* test *p* < 0.05

Mice were randomly divided in two groups, the control group (n = 5) and the sympathectomized group (n = 5). Chemical sympathectomy was performed by the intraperitoneal injection of two doses of 6-hydroxydopamine (6-OHDA; Sigma-Aldrich, St. Louis, MO) at 100 mg/kg in saline on days 0 and 1 (this dose has been reported to induce destruction of nerve endings after 3 to 5 days, with effects lasting for at least 21 days) [8]. The schedule is shown in Fig. 1. The L5178Y-R lymphoma was implanted in mice 14 days after the first 6-OHDA administration, control and sympathectomized mice were injected with 1 × 10^6^ L5178Y-R cells, in phosphate-buffered saline as vehicle, in the upper right hind leg.

#### Mice body weight and composition

Animals were weighed every two days using a digital scale; final body weight was corrected by subtracting the excised tumor weight. Body mass index (BMI) was calculated at the end of the protocol as the product of dividing the corrected body weight between the square of the nose-anus length [9]. The general state of health of experimental animals was daily monitored, including known 6-OHDA side-effects such as ptosis (upper eyelid drooping) and presence of blood in urine [10].

#### Tumor progression

Tumor volumes were determined with a digital caliper (Fisher Scientific, Hampton, NH) every third day, measuring the longitudinal tumor diameter (length) and the transverse tumor diameter (width) and using the following formula: tumor volume (mm^3^) = 1/2(length × width^2^). After mouse euthanization, tumor masses were excised and weighed.

#### Sample collection

Blood was collected by terminal cardiac puncture under surgical anesthesia induced with a mixture of ketamine 100 mg/kg and xylazine 10 mg/kg injected intraperitoneally. Cardiac puncture was performed using 1 mL syringes with a 27 G × 13 mm Luer Lock needle. Collected blood was then placed in 1.5 mL microtubes containing EDTA, and allowed to stand at 37 °C for one hour followed by centrifugation at 5000 rpm for 15 min. At the end of centrifugation, plasma was collected and stored at − 80 °C. After blood collection, animals were euthanized by cervical dislocation while still under the effects of anesthesia. Tumor masses, adipose tissue, and brain were surgically excised and stored at − 80 °C, until analysis.

#### Plasmatic cytokines

Plasma levels of IL-2, IL-4, IL-6, IFN-γ, TNF, IL-17A, and IL-10 were quantified with the Cytometric Bead Array Mouse Th1/Th2/Th17 kit from BD Biosciences (San Jose, CA), according to manufacturer instructions. Samples were processed in an Accuri 6 flow cytometer and data were analysed with the FCAP Array Software V 3.0 (Soft Flow Hungary, Ltd., Pécs, Hungary).

#### Tumor and brain TH gene expression

RNA from tumor and brain was isolated by the TRIzol^®^ Reagent (Life Technologies, Carlsbad, CA) and quantified by spectrometry using a NanoDrop 2000 (Thermo Fisher Scientific, Waltman, MA). Next, it was retrotranscribed to complementary DNA with the High Capacity cDNA Reverse Transcription Kit from Thermo Fisher Scientific. TH relative gene expression was determined using TaqMan^®^ probes (Applied Biosystems, Waltham, MA), assay number Mm00447557_m1, and GADPH, assay number Mm99999915_g1, as endogenous control. Reactions were performed in duplicate in a 7500 Real-Time PCR System (Applied Biosystems). Relative gene expression was determined by the equation 2^(−∆∆Ct)^. To calculate ΔCT, housekeeping gene mean CT from every sample was subtracted from target gene mean CT and these values were averaged for the control group. ΔΔTC was calculated for each experimental group as follows: control ΔCT was subtracted from the ΔCT of each sample from the experimental group and equation 2^−ΔΔCt^) was applied.

### Statistical analysis

The Kolmogorov–Smirnov test was used to determine data distribution and means were compared with the Student *t* test, using the statistical data package SPSS (v. 21). In all cases, *p* ≤ 0.05 was considered statistically significant.

### Results

#### Tumor progression

We observed non-significant 37% and 87% increases in tumor final volume and weight in sympathectomized mice (Table 1). Tumor volume increase was statistically significant at days 18 and 21 (*p* < 0.05) as compared with control (Fig. 1b).

**Table 1:** Body composition and tumor progression values

	Control^a^	Sympathectomized^a^
Body weight		
Gain (%)	11.06 ± 3.24	16.296 ± 2.28
Final (g)	30.08 ± 2.14	30.625 ± 1.14
BMI	0.335 ± 0.01	0.317 ± 0.00
Tumor progression		
Final volume (mm^3^)	304.46 ± 28.33	417.53 ± 20.00
Final weight (g)	0.15 ± 0.05	0.28 ± 0.16

*p < 0.05, as compared with control

^a^Data represent the mean ± SEM of 5 animals from a representative experiment

#### Mice weight and body composition

No statistically significant differences in body weight, inguinal body fat, and total adipose tissue average were observed between control and sympathectomized mice. Overall, the control and sympathectomized mice gained between 11 to 16% in body weight (Table 1). In contrast, a significant (*p* = 0.040) 60% increase in interscapular fat in sympathectomized animals was observed, as compared with control (Fig. 1b). BMI, a marker for adiposity, was not significantly different between control and sympathectomized mice (Table 1).

#### *Th* relative gene expression in tumor and brain

*Th* expression in tumor from sympathectomized mice shows a significant (*p* = 0.045) 3.3-fold decrease (Fig. 1d). In brain, a non-significant (*p* = 0.060) 6.9-fold increase was observed, compared to nerve-intact control mice.

#### Cytokine plasma levels

Cytokine levels in plasma from nerve-intact, tumor-bearing mice was marginal or absent (Table 2). On the other hand, levels of IL-4, IL-6, IFN-γ, TNF, IL-10 and IL-17A were detected in plasma from sympathectomized mice, although differences between nerve-intact and sympathectomized mice lacked statistical significance, showing high inter-individual variability.

**Table 2:** Plasmatic cytokines levels

Cytokine	Control^a^ (n = 5) (pg/ml)	Sympathectomized^a^ (n = 4) (pg/ml)
IL-4	0 ± 0	12.82 ± 17.96
IL-6	0 ± 0	79.13 ± 84.58
IFN-g	3.21 ± 7.18	8.57 ± 8.58
TNF	0 ± 0	18.06 ± 36.12
IL-10	3.53 ± 7.89	81.47 ± 84.80
IL-17A	4.08 ± 4.33	11.20 ± 6.17

*p < 0.05, as compared with control

^a^Data represent the mean ± SD of 4–5 animals from a representative experiment

### Discussion

Lymphocytes have been reported to secrete norepinephrine (NE) [3, 11], suggesting that hematopoietic and lymphoid tissue tumors might secrete catecholamines, potentially influencing surrounding nerves, other host tissues, their own function or contributing to circulating plasma levels. In the present study, the L5178Y-R lymphoma was implanted in nerve-intact and chemically sympathectomyzed male BALB/c mice (Fig. 1a). *Th* relative gene expression in brain and tumor (Fig. 1d) and plasmatic cytokines (Table 2) were quantified at day 26 post sympathectomy, whereas tumor volume and body composition were assessed during tumor progression (Fig. 1b, c).

We observed a significant increase in tumor volume of sympathectomyzed mice (Fig. 1b), whereas loss of interscapular fat was attenuated (Fig. 1c). Tumor burden has been associated with increasing lipolysis and thermogenesis activation, and surgical denervation has been shown to prevent thermogenic activation of interscapular fat tissue, preventing fat wasting in tumor-bearing cachectic mice [12]. These observations support the involvement of the sympathetic nervous system as mediator of the effects of tumor burden on lipolysis and thermogenesis, and our results are in agreement with these observations. Furthermore, treatment with beta-3 selective adrenoceptor antagonists has been reported to reduce the severity of cancer-associated cachexia, protecting interscapular fat pads from browning [12].

Regarding tumor progression, Horvathova et al. [10, 13] reported contradicting results on the effect of sympathectomy on tumor growth in a melanoma model. They found that sympathectomy delayed tumor development and significantly prolonged the survival of tumor-bearing mice. In a separate study, male C57BL/6 J mice that were sympathectomized seven days before tumor injection showed a significant increase in melanoma progression and tumor mass 20 days after tumor injection, similar to our results. The progression in tumor volume observed in our study might be explained by the temporary effect of chemical sympathectomy, because nerve endings start regenerating 21 days after the procedure, as suggested by others [8].

As for anti-tumor responses, whereas cytokine responses were mostly absent in nerve-intact mice, IL-17A, IL-6, and IL-10A were found elevated in sympathectomized tumor-bearing mice (Table 2). Although these differences were not statistically significant, there is a trend towards increased cytokine responses in sympathectomized mice. This observation is in agreement with previous reports of alterations in cytokines release in sympathectomized C57BL/C and BALB/c mice [8]. Norepinephrine released from adrenergic nerve endings is a known suppressor of immune responses; denervation interrupts NE influx, which may explain the trend observed in our study.

Regarding our main objective, we found a significant 3.3-fold reduction in *Th* gene expression in tumors from sympathectomized mice. In preliminary work, we observed that *Th* is not expressed in L5178Y-R cells cultured in vitro (data not shown), this, together with our observations, suggest that the expression of *Th* in the L5178Y-R lymphoma implanted in nerve- intact mice depends on sympathetic innervation. As seen in our results, once nerve endings are removed, levels of *Th* in tumor significantly decrease. Regarding the role of *Th* in tumor biology, it has been reported that chemical sympathectomy not only correlated with smaller tumors, but also significantly increased TH, neuropeptide Y, and glucocorticoid receptor gene expression in tumors from male C57BL/6 J mice [13]. Our data showed larger tumors with decreasing *Th* expression after sympathectomy. Taken together, the results of Horvathova et al*.* [13] and our data both point to a relationship between *Th* expression and tumor size.

Furthermore, the 6.9-fold increase in brain *Th* gene expression, although not statistically significant (*p* = 0.06), follows a trend that has been observed in other studies and has been explained as a potential compensatory mechanism for the loss of adrenergic innervation [14]. It has been observed that 6-OHDA denervation suppresses endogenous NE production and uptake by the hippocampus within 5 days of 6-OHDA administration. However, at 21 days, hippocampal TH and NE synthesis significantly increases, which may suggest brain plasticity and adaptive response [14], this may explain our finding of increased *Th* gene expression in brain. Moreover, the activation of the central nervous system by sympathectomy has been previously reported [15].

Our results do not support our working hypothesis of the L5178Y-R lymphoma expressing *Th* during its progression. Further work is necessary to expand our understanding of the relationship between central adrenergic activation and tumor burden.

## Limitations

Our experimental design lacked controls for the effect of tumor burden on cytokine responses and animals’ adiposity. Data from tumor-free animals could also have been informative of potential tumor burden effects on brain *Th* expression in this lymphoma model. In this regard, lacking norepinephrine measures in tumor, brain, and plasma limited the interpretation of the increased expression of *Th* in brain. Sample size was an additional limitation.

## Data Availability

Raw data and materials are available from the corresponding author at request.

## Abbreviations

6-OHDA: 6-Hydroxydopamine
BMI: Body mass index
NE: Norepinephrine
PCR: Polymerase chain reaction
TH: Tyrosine hydroxylase
